# Targeted Delivery of Narrow-Spectrum Protein Antibiotics to the Lower Gastrointestinal Tract in a Murine Model of *Escherichia coli* Colonization

**DOI:** 10.3389/fmicb.2021.670535

**Published:** 2021-10-14

**Authors:** Nuria Carpena, Kerry Richards, Teresita D. J. Bello Gonzalez, Alberto Bravo-Blas, Nicholas G. Housden, Konstantinos Gerasimidis, Simon W. F. Milling, Gillian Douce, Danish J. Malik, Daniel Walker

**Affiliations:** ^1^College of Medical, Veterinary and Life Sciences, University of Glasgow, Glasgow, United Kingdom; ^2^Chemical Engineering Department, Loughborough University, Loughborough, United Kingdom; ^3^Department of Biochemistry, University of Oxford, Oxford, United Kingdom

**Keywords:** antibiotic resistance, bacteriocins, drug delivery, hydrogels, membrane emulsification, microbiome engineering

## Abstract

Bacteriocins are narrow-spectrum protein antibiotics that could potentially be used to engineer the human gut microbiota. However, technologies for targeted delivery of proteins to the lower gastrointestinal (GI) tract in preclinical animal models are currently lacking. In this work, we have developed methods for the microencapsulation of *Escherichia coli* targeting bacteriocins, colicin E9 and Ia, in a pH responsive formulation to allow their targeted delivery and controlled release in an *in vivo* murine model of *E. coli* colonization. Membrane emulsification was used to produce a water-in-oil emulsion with the water-soluble polymer subsequently cross-linked to produce hydrogel microcapsules. The microcapsule fabrication process allowed control of the size of the drug delivery system and a near 100% yield of the encapsulated therapeutic cargo. pH-triggered release of the encapsulated colicins was achieved using a widely available pH-responsive anionic copolymer in combination with alginate biopolymers. *In vivo* experiments using a murine *E. coli* intestinal colonization model demonstrated that oral delivery of the encapsulated colicins resulted in a significant decrease in intestinal colonization and reduction in *E. coli* shedding in the feces of the animals. Employing controlled release drug delivery systems such as that described here is essential to enable delivery of new protein therapeutics or other biological interventions for testing within small animal models of infection. Such approaches may have considerable value for the future development of strategies to engineer the human gut microbiota, which is central to health and disease.

## Introduction

The global rise in infections attributed to antibiotic-resistant Gram-negative bacteria poses a serious public health threat. Foremost among the Gram-negative pathogens are *Escherichia coli*, *Klebsiella pneumoniae* for which clinical isolates with extensive drug resistance, including to antibiotics of last resort such as carbapenems, are frequently encountered (Logan and Weinstein, 2017). Together with other generally drug-resistant bacterial species, such as Enterococci, these bacteria can be problematic in the hospital environment as they can dominate the microbiota of patients treated with broad spectrum antibiotics and may subsequently cause serious nosocomial infections (Ravi et al., 2019). In addition, Gram-negative bacteria are frequently present at elevated levels in dysbiotic microbiota associated with non-infectious chronic disease, where proteobacteria such as *E. coli* are generally overrepresented. For example, increased levels of *E. coli*, specifically adherent-invasive *E. coli* (AIEC), are associated with Crohn’s disease (CD), an incurable form of inflammatory bowel disease (Darfeuille-Michaud et al., 2004; Lloyd-Price et al., 2019). New strategies are therefore required to target problematic gram-negative bacteria within the human microbiota.

Narrow-spectrum protein bacteriocins are emerging as promising alternative antibiotics and could be utilized to target specific pathogenic bacteria in the complex microbial community of the human microbiota, sparing the key eubiotic organisms (Brown et al., 2015; McCaughey et al., 2016; Six et al., 2020). The best studied of the protein bacteriocins are the colicins, which specifically target *E. coli* and strains of other closely related bacterial species (Cascales et al., 2007). The narrow killing spectrum of colicin-like bacteriocins is dictated by the numerous protein–protein interactions involved in the import of these 40–70kDa toxins (Kleanthous, 2010; Behrens et al., 2019). Colicins, and other protein bacteriocins, deliver a single cytotoxic activity that either depolarizes the inner membrane, hydrolyses DNA or RNA in the cytoplasm or abolishes cell wall biosynthesis in the periplasm. Colicin E9, for example, is a non-specific DNase and colicin Ia forms voltage-gated ion-conducting channels in the cytoplasmic membrane (Pommer et al., 2001; Jakes and Finkelstein, 2010).

Bacteriocins are deployed naturally by both commensal and pathogenic organisms to augment niche colonization through the displacement of closely related bacteria. Colicins have been shown to play a direct role in the success of pathogenic *Salmonella* competing against commensal *E. coli* in enteric blooms and were a contributory factor in the success and spread of *Shigella sonnei* in Vietnam (Holt et al., 2013; Nedialkova et al., 2014). However, although these studies demonstrate that colicins can be successfully deployed within the environment of the gastrointestinal (GI) tract when produced *in situ*, it has also been demonstrated that colicins are highly susceptible to degradation by the proteases deployed in the stomach and small intestine to digest proteins (Schulz et al., 2015). Therefore, to utilize purified bacteriocins as orally dosed therapeutics, choice of a delivery formulation that protects against proteolysis in the upper GI tract but allows release in the lower GI tract will be essential.

In this work, we explore the potential of orally dosed colicin E9 and Ia to target bacteria in the lower GI tract. To protect colicins during transit through the upper GI tract and to enable controlled release of high doses of active agent in the lower GI tract, we encapsulated purified colicin E9 and Ia in pH-responsive microcapsules. Oral dosing demonstrated that active encapsulated colicins can be delivered to the lower GI tract in a murine *E. coli* colonization model.

## Materials and Methods

### Bacterial Strains and Purification of Recombinant Colicins

For colicin overexpression, plasmids based on pET21a encoding the genes for the colicin E9-Im9 complex with a C-terminal His6-tag on the immunity protein and colicin Ia carrying a C-terminal His6-tag were transformed into *E. coli* BL21 (DE3) pLysS (Promega). Cells were grown in Luria–Bertani Broth media (LB) supplemented with ampicillin (100μg ml^−1^), until the OD_600_ reached 0.6. The cultures were induced with 0.1mM IPTG β-D thiogalactopyranoside (IPTG) for 20h at 28°C to express colicin Ia and with 1mM IPTG for 3h at 37°C to express colicin E9. After induction, cells were harvested by centrifugation (5,000 rpm for 15min) at 4°C. Cell pellets were re-suspended in lysis buffer (50mM Tris, 200mM NaCl pH 7.5) supplemented with DNAse I (1μg/ml, Sigma-Aldrich), lysozyme (1mg/ml, Sigma-Aldrich), and protease inhibitor tablet (cOmplete™, EDTA-free Protease Inhibitor Cocktail, Sigma-Aldrich) and lysed by sonication for 15cycles (15s on, 45s off). Cell debris was removed by centrifugation (18,000 rpm for 20min at 4°C), and supernatants were filtered through 0.22μm syringe filters and applied to a His trap™ HP column (GE healthcare). The columns were washed using a modified lysis buffer containing 20mM imidazole, followed by a 50mM imidazole wash. Finally, the proteins were eluted with 500mM imidazole. Colicins isolated by nickel affinity chromatography were concentrated and further purified by size exclusion chromatography, Superdex HiLoad 26/600 Superdex 200 pg. column (GE Healthcare), in 50mM Tris-HCl 200mM NaCl pH 7.5 solution. The protein concentrations were determined by ultraviolet absorption at 280nm, using the extinction coefficient of 0.807M^−1^ cm^−1^ for E9-Im9 and 0.855M^−1^ cm^−1^ for colicin Ia.

To determine colicin killing activity and for *in vivo* experiments a spontaneous streptomycin resistant mutant of the AIEC reference strain LF82, an ileal CD mucosa-associated isolate (Darfeuille-Michaud et al., 2004) was selected following treatment with this antibiotic and transformed with the p16S*lux* plasmid which contains the erythromycin resistance cassette (*erm*AM). This strain, *E. coli* LF82StrR, was grown on LB agar plates, or in LB broth with shaking at 37°C with the addition of ampicillin (100μg ml^−1^) and erythromycin (500μg ml^−1^). Before infection, bacteria from overnight cultures were diluted 1:100 in fresh media and grown to an OD_600_ of 0.65, which is equivalent to approximately 1×10^9^ c.f.u/ml. Subsequently, cultures were harvested, washed and resuspended in PBS. For *in vivo* experiments, counts of challenge dose were plated before and after infection to determine viable bacterial numbers.

### Colicin Killing Activity Assay

Colicin killing activity was determined using a spot test. Briefly, aliquots (100μl) of overnight cultured LF82StrR were transferred into 5ml of soft agar (0.8% w/v); the mixture was then poured onto an LB agar plate (1.5% w/v) supplemented with 100mM bipyridine. An aliquot (10μl) of colicin (1mg/ml) was spotted onto the soft agar layer seeded with cultured bacteria. After overnight incubation at 37°C, plates were visualized for colicin sensitivity observed as clear zones of lysis of the overlaid strains. To evaluate the hydrogel encapsulated colicins, 10mg of the colicin E9 or Ia hydrogels were resuspended with 1ml of PBS (pH 6.8) and incubated for 24h. An aliquot (10μl) of hydrogels was spotted onto the soft agar layer seeded with cultured bacteria. After overnight incubation at 37°C, plates were visualized for colicin sensitivity observed as clear zones of lysis of the overlaid strains.

### Murine *E. coli* LF82 Colonization Model

All procedures were performed in strict accordance with the Animals (Scientific Procedures) Act 1986 with specific approval granted by the Home Office, United Kingdom (PPL60/8797; P64BCA712). Food and water were provided *ad libitum,* and animals were kept at a constant room temperature of 20–22°*C with a 12h light/dark cycle*. Eight- to ten-week-old, pathogen-free C57/BL6 female mice (The Jackson Laboratory, Envigo) were pre-treated by a single oral administration of the broad-spectrum antibiotic streptomycin (20mg, intragastric per mouse) to disrupt normal resident bacterial flora in the gastrointestinal tract (Wadolkowski et al., 1988). Before the administration of the LF82StrpR strain, fecal pellets were collected, plated on LB agar plates with streptomycin (100μg ml^−1^) and erythromycin (500μg ml^−1^) and incubated at 37°C. No colonies were detected in any of the streptomycin treated mice. Mice were then orally challenged with approx. 1×10^9^ cfu of LF82StrpR or 0.1ml PBS (control group) 24h post-antibiotic treatment. LF82StrpR colonization was monitored by analysis of bacterial recovery on selective LB plates of fresh fecal material collected from individual animals.

### Colonization Evaluation in Stool and Tissues

Fresh stool samples were collected from infected mice 1, 2, 3, and 4days post-LF82 challenge to determine bacterial fecal shedding. Fecal pellets standardized to a concentration of 100mg ml^−1^ in PBS were homogenized and serial 10-fold dilutions performed. About 10μl of diluted samples was plated on Eosin Methylene Blue (EMB) differential medium agar containing ampicillin (100μg ml^−1^) and erythromycin (500μg ml^−1^) and incubated at 37°C overnight. Ileum, caecum, and colon samples were collected 4days post-infection in cold PBS at necropsy and homogenized using Tissue Master 125 Homogenizer (OMNI International). Homogenate tissues were serially diluted and plated on EMB agar containing ampicillin (100μg ml^−1^) and erythromycin (500μg ml^−1^). Colonies were counted after 24–48h of incubation at 37°C and expressed as c.f.u. per gram of tissue.

### Delivery of Colicin by Direct Injection in *E. coli* LF82 Colonized Mice

The above murine colonization model was used to assess the efficacy of a colicin E9/Ia in reducing LF82 levels in the lower GI tract after a single treatment administration of colicin by direct injection in the caecum. Four days after LF82 challenge, mice were treated with 50μl of a combination of E9 and Ia (0.5mg ml^−1^) or 50μl of PBS (control group), directly injected into the caecum after laparotomy. Animals were maintained under inhalation anesthesia with isoflurane (Abbott Labs, Abbott Park, IL) during surgery and were allowed to fully recover. Three hours post-treatment, mice were killed and LF82StrpR colonization levels of the different regions of the GI tract (ileum, ceacum, and colon) and fecal content were assessed. For bacterial counts, tissue samples were washed thoroughly with PBS prior to homogenization to eliminate fecal content and non-adhered bacteria. For fecal samples, fecal pellets from the colon were homogenized.

### Treatment of *E. coli* LF82 Colonized Mice With Encapsulated Colicins

C57/BL6 animals were treated as described above for the murine colonization model, with the addition of dextran sulfate sodium (DSS) which was added to drinking water at a concentration of 2.5% 3days before the LF82 challenge. This concentration of DSS was maintained for 3days (renewed daily) and caused mild symptoms of colitis which improved LF82 adherence to the mucosal layer. Three days after LF82StrpR challenge, mice were orally treated with two doses of colicins per day, delivered 7h apart, with 200μl of hydrogel particles containing colicins E9 and Ia (0.5mg each) or control hydrogel particles by gavage in a delivery buffer (sodium acetate buffer, 2% Tween 20, 50% glucose, pH 3.8). Animals were treated for a total of 3days. On the fourth day, mice were culled by cervical dislocation and LF82StrR colonization levels of the different parts of the GI tract and fecal content were assessed as detailed above.

### Measurement of Gastrointestinal Luminal pH

The impact of exposure of gut tissue to the low pH of the delivery buffer was determined *ex vivo* following aseptically extraction of the luminal contents from the small intestine, caecum, and colon. Tissue and luminal contents were then placed in 1ml of sterile PBS, delivery buffer (200mM sodium acetate buffer, 2% Tween 20, 50% glucose, pH 3.8), or sterile water. Organs and luminal contents were incubated, and pH measurements were acquired 2h later. Data represent pH values (mean±SD) in each buffer.

### Chemical Reagents Used to Prepare Hydrogel Microcapsules With Encapsulated Colicins

Water-in-oil emulsion production employed a continuous (oil) phase composed of Miglyol 840 (Safic Alcan, Warrington, United Kingdom), a propylene glycol diester of saturated plant fatty acids and Polyglycerol polyricinoleate (PGPR, Aston Chemicals Ltd., Aylesbury, United Kingdom), an emulsifier made from glycerol and fatty acids. The aqueous dispersed phase contained a pH responsive polymer, Eudragit L 100-55 (Evonik, Germany), which is an anionic copolymer based on ethyl acrylate and methacrylic acid. Medium viscosity alginate (Sigma-Aldrich, Dorset) was added to the dispersed phase. Sodium chloride, sodium hydroxide, and p-toluenesulfonic acid (pTSA) were all purchased from Fisher Scientific, Loughborough, United Kingdom.

### Production of Encapsulated Colicins in Microcapsules Using Membrane Emulsification

The continuous (oil) phase was produced by preparing a solution of miglyol and castor oil (9:1, respectively) with the addition of 5% PGPR to lower the interfacial tension between water and oil. The dispersed phase was composed of 10% (w/v) Eudragit polymer L100-55 dissolved in an alkaline solution, typically produced in 40ml batches with 4ml of 4M NaOH, 36ml de-ionized water (dH2O), and 4g L100-55. This solution was mixed with a magnetic stirring bar at room temperature until the solution appeared clear. Subsequently, pre-weighed alginate powder was added at a final concentration of 1% (w/v) and mixed with a magnetic stirring bar overnight or until completely dissolution. Immediately before the membrane emulsification process commenced, colicin was added (typically E9: 100mg, Ia: 80mg). The solution was mixed gently using a magnetic stirrer for 5min to disperse the colicin in the polymer solution. Each colicin was encapsulated separately to allow accurate enumeration of the amount of each colicin administered to the animals.

**Figure 1:**
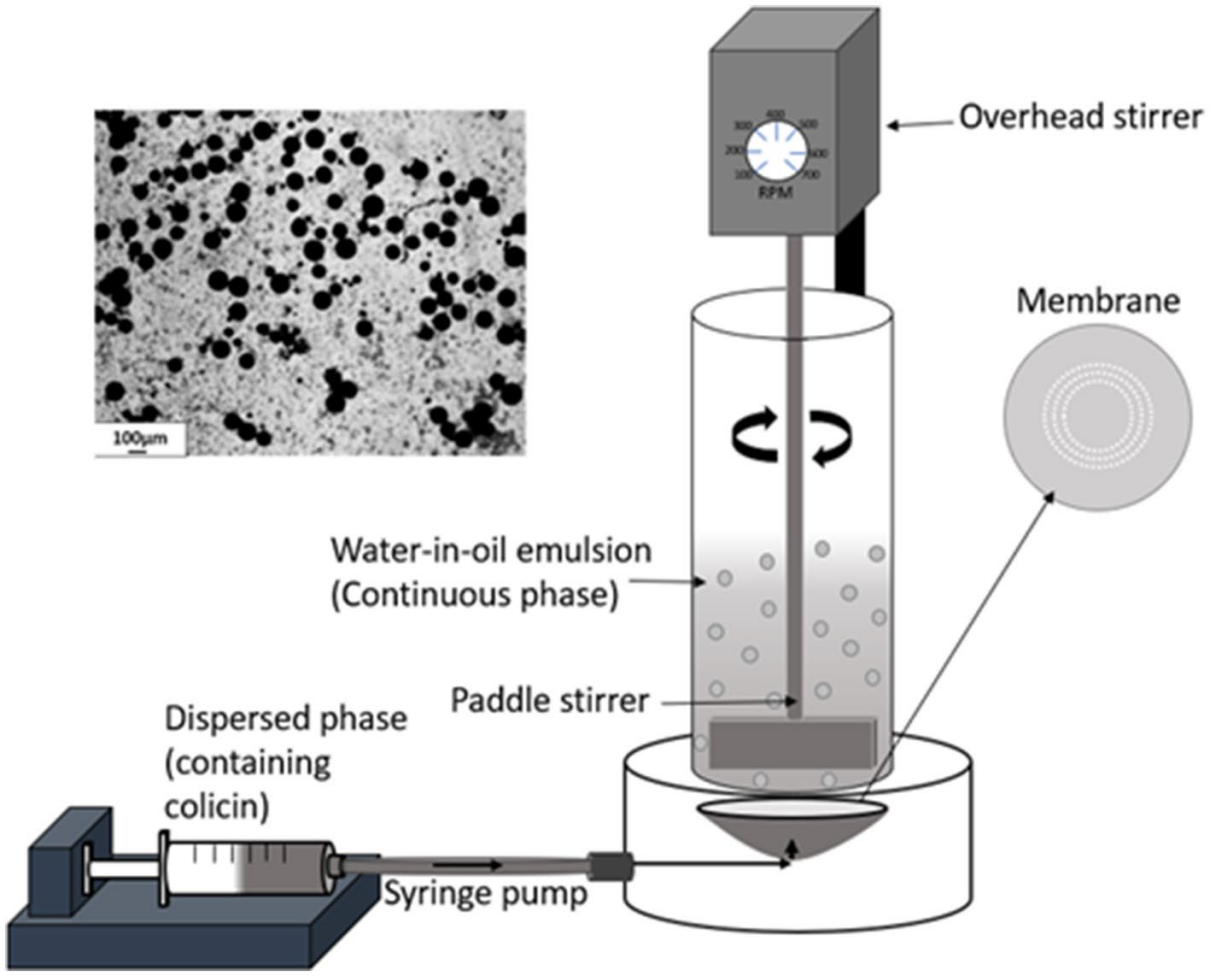
Schematic representation of the membrane emulsification process. The aqueous phase containing the dissolved colicins in the polymer formulation was pumped through a microporous membrane. The stainless-steel membrane had 40μm uniform circular pores arranged in a ring format located at an optimal radial position where the rotating paddle stirrer provides maximum surface shear for aqueous droplet detachment. At low liquid flow rates, interfacial forces dominate, the drop grows before finally detaching from the membrane surface due to the surface shear caused by the dispersed phase flowing across the membrane surface and the droplets are carried away into the bulk continuous oil phase. The image (top left) shows the prepared microcapsules after exposure to TSA and alginate cross-linking.

Aqueous colicin containing droplets were produced as a water-in-oil (W/O) emulsion using a membrane emulsification dispersion cell LDC-1 (Micropore Technologies Ltd., Redcar, United Kingdom). A stainless-steel membrane was utilized with circular, uniformly spaced 40μm micropore arrays. Initially, the membrane was coated in 1H,1H,2H,2H-Perfluorodecyltriethoxysilane (Sigma-Aldrich, Gilingham, United Kingdom) resulting in a hydrophobic surface to prevent water droplets from spreading on the membrane surface. The dispersed phase was used to fill the cavity below the membrane using a syringe pump (Harvard Apparatus United Kingdom, Kent). About 50ml of continuous phase was added above the membrane into the cylindrical glass chamber. A paddle blade stirrer was used to create shear on the membrane surface using a controlled rotation rate of 250 revolution per minute (rpm). About 5ml of the dispersed phase was then pumped upwards through the membrane at a flow rate of 25ml h^−1^ to produce a W/O emulsion in the glass chamber (Figure 1).

After the entire volume of the dispersed phase had passed through the membrane and into the oil phase, protonation of the polymer was carried out to precipitate the polymer resulting in formation of the microcapsules. The water-in-oil emulsion was added to acidified oil (Miglyol with 0.05M pTSA and 5% PGPR) in an excess volume. The emulsion in acidified oil was placed into a beaker and stirred using axial mixing at 100rpm for 6h at a controlled temperature of 25°C using a water bath.

After the TSA step, the W/O emulsion was added to hexane. The amount of hexane to emulsion proportion was 50:50 (v/v) in a beaker. The TSA-treated microcapsules formed a precipitate at the bottom of the beaker. The hexane was then discarded and the microcapsules washed with 2% Tween-20 in deionized water (pH 4). The sample was gently stirrer at 60rpm using a magnetic stirrer throughout this resuspension step to produce a well-dispersed sample and to avoid formation of aggregates. 1M CaCl_2_ was then added to the solution to cross-link the alginate (final working concentration 0.1M), then mixed using a three-bladed impeller at 100rpm for 1h. The microcapsules were then washed three times with 2% Tween solution (pH 4) and stored in 10ml of this solution in a refrigerator (4°C).

### Characterization of the Particle Size Distribution of the W/O Emulsion and the Final Cross-Linked Microcapsules

Throughout the encapsulation process, samples were imaged using a high-speed camera (Micro C100 Phantom Ametek, United Kingdom) which was connected to a microscope (Nikon Eclipse E200). A x 10 magnification lens was used to view each sample, and these images were captured through connection to a laptop and use of the Phantom Camera Control software (PCC 3.1). The size distribution of the droplets and microcapsules were measured using a Coulter LS series 130 instrument (Beckmann Coulter Inc). About 15ml of miglyol +5% PGPR was placed into the coulter sample chamber. Typically, 100μl of emulsion was added until the obscuration level measured was between 8 and 12%. For microcapsule particle size characterization, 15ml of 2% (v/v) tween in deionized water (pH 4) was used as the sample diluent and 100μl of suspended particles was added until the correct obscuration level was reached. Three repetitions of each measurement were taken and results averaged to produce the final size distribution curves (Supplementary Figure S1).

### Measuring the Activity of Encapsulated Colicin in Eudragit L100-55 Hydrogel Microcapsules

To test the colicin activity of colicin encapsulated in hydrogel microcapsules and evaluate the release kinetics, 0.1g of the hydrogels (containing either E9 or Ia) was weighed and exposed to 1ml of Sorensen’s buffer, pH 5.5. Samples were taken over a period of 2h by removing 10μl of supernatant and serially diluting with 90μl of sterile Sorensen’s buffer to measure the release kinetics of colicin from the capsules. The absorbance (OD_280nm_) was measured using a UV spectrophotometer. Using the extinction coefficient and quartz cuvette path length, the colicin concentration was enumerated at each time point. The viability of the colicin after release from the microparticles was confirmed using the double-layer agar method, with *E. coli* LF82 as the indicator strain, and compared to colicin stocks before encapsulation. This allowed assessment of colicin activity following encapsulation using the microcapsule production process and that the released colicin retained its *E. coli* killing potency. The acid stability of the colicin containing hydrogels was tested using the same method indicated above, with exposure to 0.2M NaCl (pH 2.5) for 2h simulating exposure to mouse gastric fluid before colicin release from the capsules in Sorensen’s buffer (pH 5.5).

### Sample Preparation for the Ion Microscopy

Critical point drying (CPD) and freeze-drying methods were used to prepare the hydrogel samples for ion microscopy. The particles were freeze-dried (VirTis Wizard 2.0, SP Scientific, New York, United States) for 24h at 50Pa pressure and −20°C. Dried powder was applied directly on the carbon tape, which was attached to the sample stub. To analyze the morphology of the hydrogel particles, both freeze-dried and critical-point-dried hydrogels were examined with ion microscopy. Zeiss Orion NanoFab (University of Jyväskylä) with He^+^ beam and acceleration voltage 35kV, 0.20 pA current, 32 line averages, and 1μs dwell time was used for He^+^ imaging. For cutting, an about 20-pA Ne^+^ beam with 10kV acceleration voltage was used. Milling was carried out using a 45 degrees tilted angle by setting the reduced raster scan rectangle over the area to be removed and scanning until the material disappeared. After cutting, the sample stage was rotated 180° and the cross section was imaged with a He^+^ beam. Flood gun charge compensation was used during both milling and imaging.

### Statistics

Data are expressed as means and SD. Due to small sample sizes, nonparametric tests were used for analysis. Two-tailed Mann–Whitney U tests with a significance threshold of *p*≤0.05 were used to analyze the specific sample pairs for significant differences. Mice colonization data are represented using Tukey’s box-and-whisker plot. All statistical tests were performed with GraphPad Prism software, version 8.0c. All mice, including outliers, were included in the statistical analysis.

## Results

### *In vivo* Colicin Activity After Direct Administration to the Lower GI Tract

Colicins have been shown to be highly sensitive to proteolytic cleavage in conditions found in the stomach and small intestine (Schulz et al., 2015). However, little is known about their stability and activity in the lower GI tract. To determine if colicins retain killing activity against *E. coli* in the environment of the lower GI tract, a combination of colicin IA and E9 were injected directly, during laparotomy, into the caecum of mice pre-colonized with the adherent-invasive *E. coli* strain LF82. This model is widely used in the study of CD and enables relatively stable colonization of the lower GI tract, including the colon and ileum, which are the major sites of AIEC colonization and inflammation in this condition (Darfeuille-Michaud et al., 2004). Colicin E9 and Ia were selected for testing since they show broad activity against a panel of AIEC and commensal strains isolated from CD patients and healthy controls, respectively.

**Figure 2:**
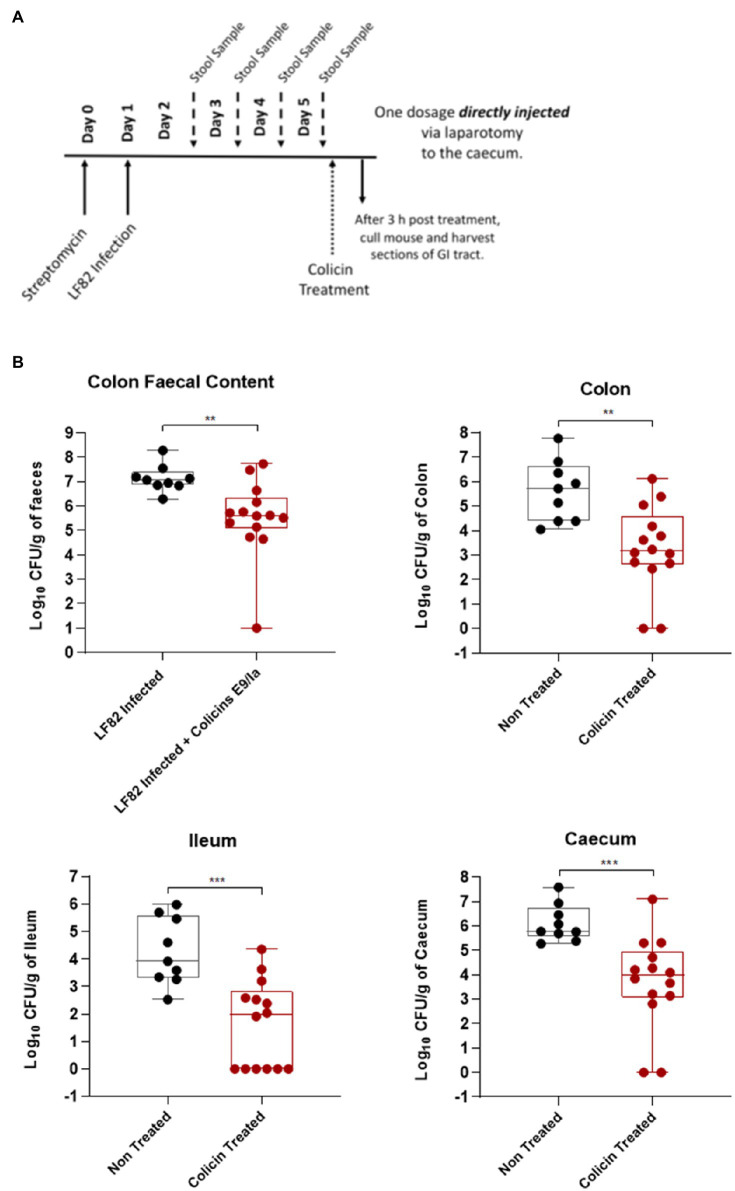
Direct administration of a colicin cocktail reduces levels of LF82 colonization of infected mice. An *Escherichia coli* LF82StrpR murine infection model was used to assess the efficacy of a colicin cocktail treatment directly administered to the caecum *via* laparotomy. Four days after LF82StrpR challenge, mice were treated with the administration *via* laparotomy of 50μl of a combination of E9 and Ia (0.5mg ml^−1^ each) or 50μl of PBS (control group). **(A)** Experimental scheme. **(B)** Levels of LF82StrpR strain in both control (black, *n*=10) and colicin treated (red, *n*=14) groups for the different sections of the GI tract. Statistical analysis was carried out for each subset using a Mann–Whitney test between LF82StrpR infected and colicin-treated groups. ^**^p<0.002, ^***^p<0.0004.

After streptomycin treatment to disrupt the endogenous microbiota, mice were infected with LF82 and 4-days post-infection were treated with a single dose of colicin E9/IA or PBS for the control group (Figure 2A). Three hours post-treatment mice were killed, and AIEC colony forming units were determined in tissue of the ileum, caecum, and colon and fecal content of the colon. Colicin E9/IA administered by direct injection resulted in significant reductions in LF82 levels in the ileum (1.9 log units), caecum (1.7 log units), colon (1.5 log units), and in the fecal content (1.5 log units), relative to PBS-treated controls (Figure 2B). Thus, a single dose of colicin E9/Ia is able to reduce *E. coli* levels in the lower GI tract. These data indicate that if colicins can be formulated to protect them from the high levels of proteolytic activity associated with the stomach and small intestine, then a highly targeted killing activity of *E. coli* could be achieved through delivery of these protein antibiotics to the lower GI tract.

### Colicin Formulation for Lower GI Tract Delivery

To formulate colicins for lower GI tract delivery, we attempted to encapsulate colicin E9 and Ia in pH-responsive hydrogel microcapsules consisting of a commercially available mixture of synthetic polymer containing ethyl acrylate and methacrylic acid monomers combined with seaweed derived alginate biopolymer (Supplementary Figure S2). This technique has previously been used to formulate enteric bacteriophages for gastrointestinal applications affording protection from acidic pH and enzymatic stresses. Controlled release relies on dissociation of carboxylic acid groups when the pH rises above 5 (Supplementary Figure S2). Based on our own measurements of pH from mouse stomach tissue samples (*in vivo* studies reported below), these range from pH 3.8–5.4 depending on the diluent medium used. In contrast, the pH of the small intestine, caecum, and colon tissue samples was above pH 5.5 when either PBS or water was used as the diluent. Using the mildly acidic delivery buffer (pH 3.8) as the diluent resulted in considerably lower measured pH values. Freeze-dried microparticles appeared as spheres with a smooth and uniform surface (Supplementary Figure S2C, left). CPD-dried microparticles displayed a sponge-like surface (Supplementary Figure S2C, right). The internal structure was found to be porous with an interconnected network of much smaller pores.

**Figure 3:**
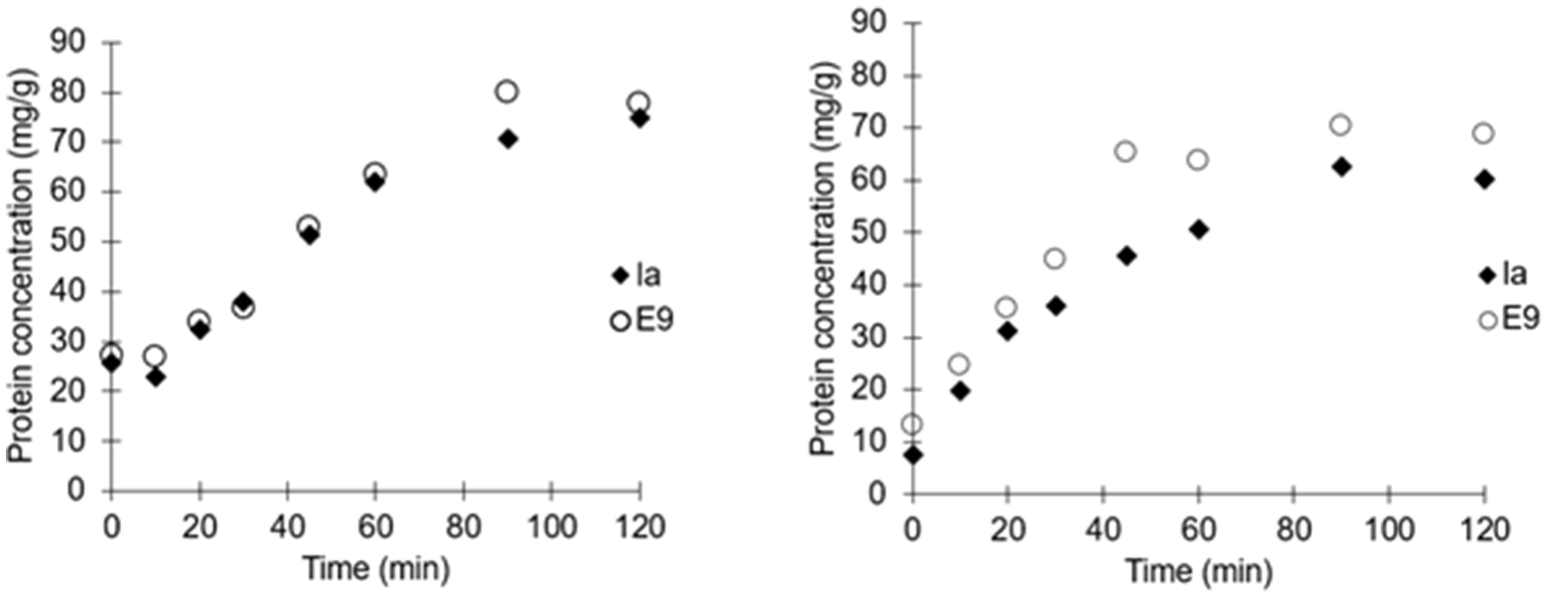
Release kinetics of individually encapsulated colicins Ia and E9. Microcapsules exposed to simulated intestinal fluid buffer (SIF) at pH 5.5 (Left). Release kinetics of individually encapsulated colicins Ia and E9 in microcapsules exposed to simulated gastric fluid (SCF; pH 2.5) for 2h followed by release in SIF at pH 5.5 (Right).

Colicins E9 and Ia were encapsulated by membrane emulsification yielding small microcapsules around 100μm in diameter, which when suspended in a buffer were suitable for delivery to mice *via* oral gavage using a 20 gauge gavage tube (Figure 1). The dispersed phase alginate concentrations were varied to determine the concentration with optimal viscosity levels for controlled production of 100μm beads (Supplementary Figure S3). Initial analysis confirmed 1% (w/v) alginate produced approximately 100μm microcapsules, whereas 0.5% (w/v) alginate produced mean diameters of ~35μm with high CV values of ~80%. 2% (w/v) alginate caused membrane fouling and reduced encapsulation efficiency. The size distribution of the emulsion droplets and the resulting cross-linked hydrogel microcapsules were similar although a slight shrinkage in the size of the microparticles was observed upon gelation (Supplementary Figure S1). The yield of the encapsulated colicins in the hydrogel microcapsules was high, with no measurable loss in activity due to the encapsulation process as measured following release in simulated intestinal fluid at pH 5.5 (Figure 3). The polymer L100-55 was selected due to its pH triggered dissolution at solution pH 5 and above. The amount of encapsulated E9 released at pH 5.5 from the hydrogel capsules was ~80mg/g, whereas that of Ia was ~70mg/g which was almost 100% of the colicin added to the polymer solution for fabrication of the microcapsules (Figure 3). The release kinetics were similar for the two batches of microcapsules with 50% of the encapsulated colicin released in the first 30min and over 90% released within 90min of the hydrogel microcapsules being exposed to the pH 5.5 buffer. Exposure of the hydrogels to acidic buffer (pH 2.5) mimicking harsh simulated gastric fluid (SGF) conditions in the mouse stomach for 2h resulted in a modest reduction in the activity of the encapsulated colicin in the microcapsules with released amounts falling to ~70mg/g for E9 and ~60mg/g for Ia. Over 90% of the encapsulated colicin was still released from the acid exposed microcapsules within 90min. The *in vitro* data confirm the colicin was encapsulated within the alginate/Eudragit matrix. As the microcapsules were exposed to acidic buffer and the colicin retained lytic activity after capsule dissolution as the pH increased, this verifies the colicin resides inside of the microcapsule. The hydrogel capsules used for the *in vivo* experiments were not pre-exposed to SGF.

**Figure 4:**
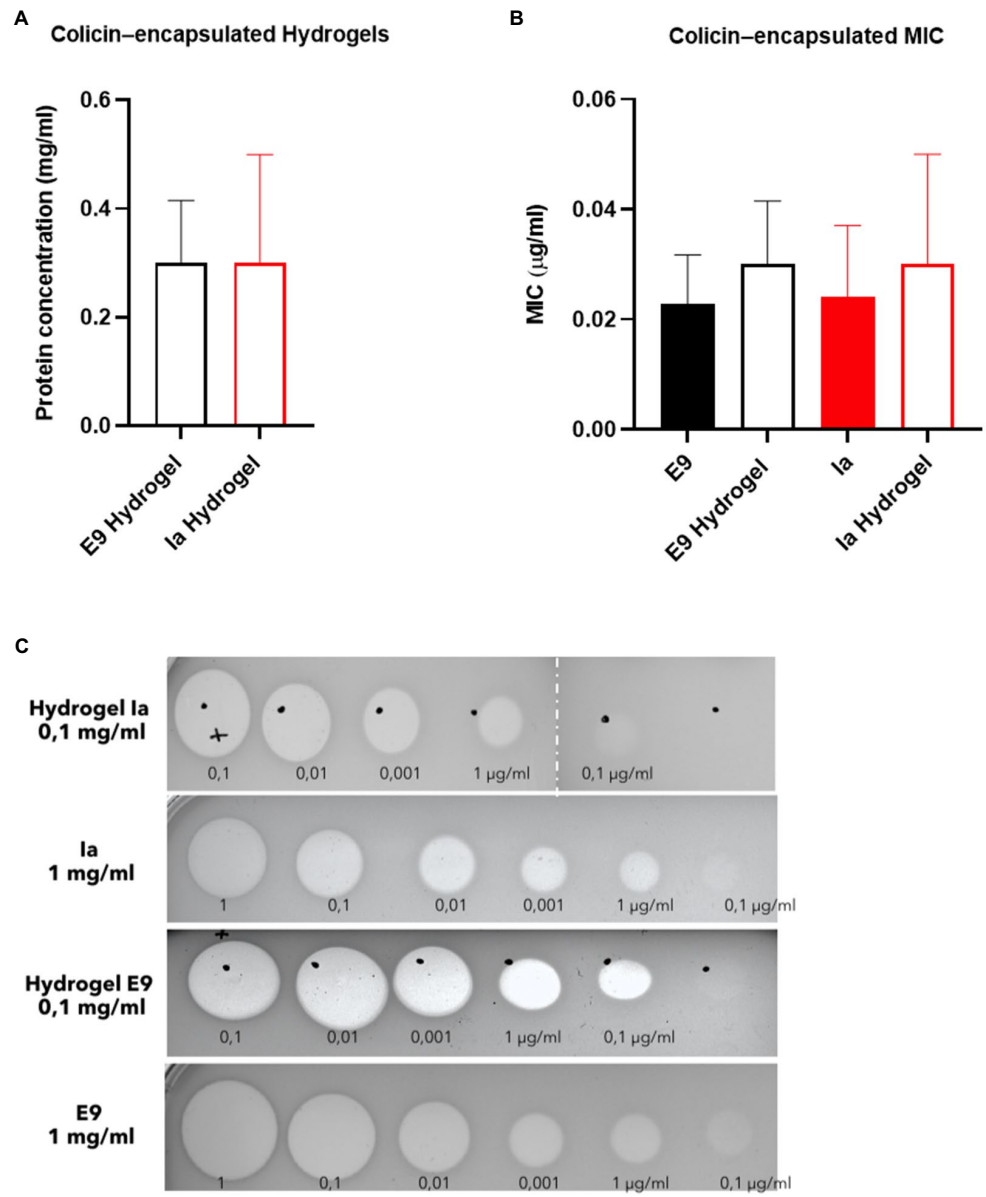
Killing activity of hydrogel-encapsulated colicins. **(A)** Levels of encapsulated protein (mg/ml) after suspension. **(B)** Minimum inhibitory concentration (MIC) and **(C)** inhibition of growth of *E. coli* LF82StrpR by colicins E9 and Ia and hydrogel-encapsulated colicin E9 and Ia. Clear zones indicate cell death. Data represent four different experiments (mean±SD). Statistical analysis was carried out for each subset using a Mann–Whitney test between the control protein and the hydrogel particles and no significant differences were found. Hydrogel microcapsules that did not contain encapsulated colicins showed no bactericidal activity.

Hydrogel microcapsules were shipped in a cool box from Loughborough (United Kingdom) to Glasgow (United Kingdom) for testing in the *in vivo* mouse model and were immediately stored in the fridge upon arrival. Samples were evaluated *in vivo* within a week of manufacture. A significant reduction in the activity of colicin was observed during this transportation and storage period. Measured protein values prior to administration of the capsules to the animals showed around 30mg/g for both Ia and E9 given that 10mg of capsules was dissolved in 1ml of buffer, and this resulted in a measured concentration of 0.3mg/ml (Figure 4A). The killing activity of the released colicins from the hydrogels was similar to that of free colicin and the released colicin showing activity on plates with clear zones indicating cell death (Figures 4B,C).

### *In vivo* Colicin Activity After Repeat Dose Oral Administration of Colicin Containing Hydrogels

**Figure 5:**
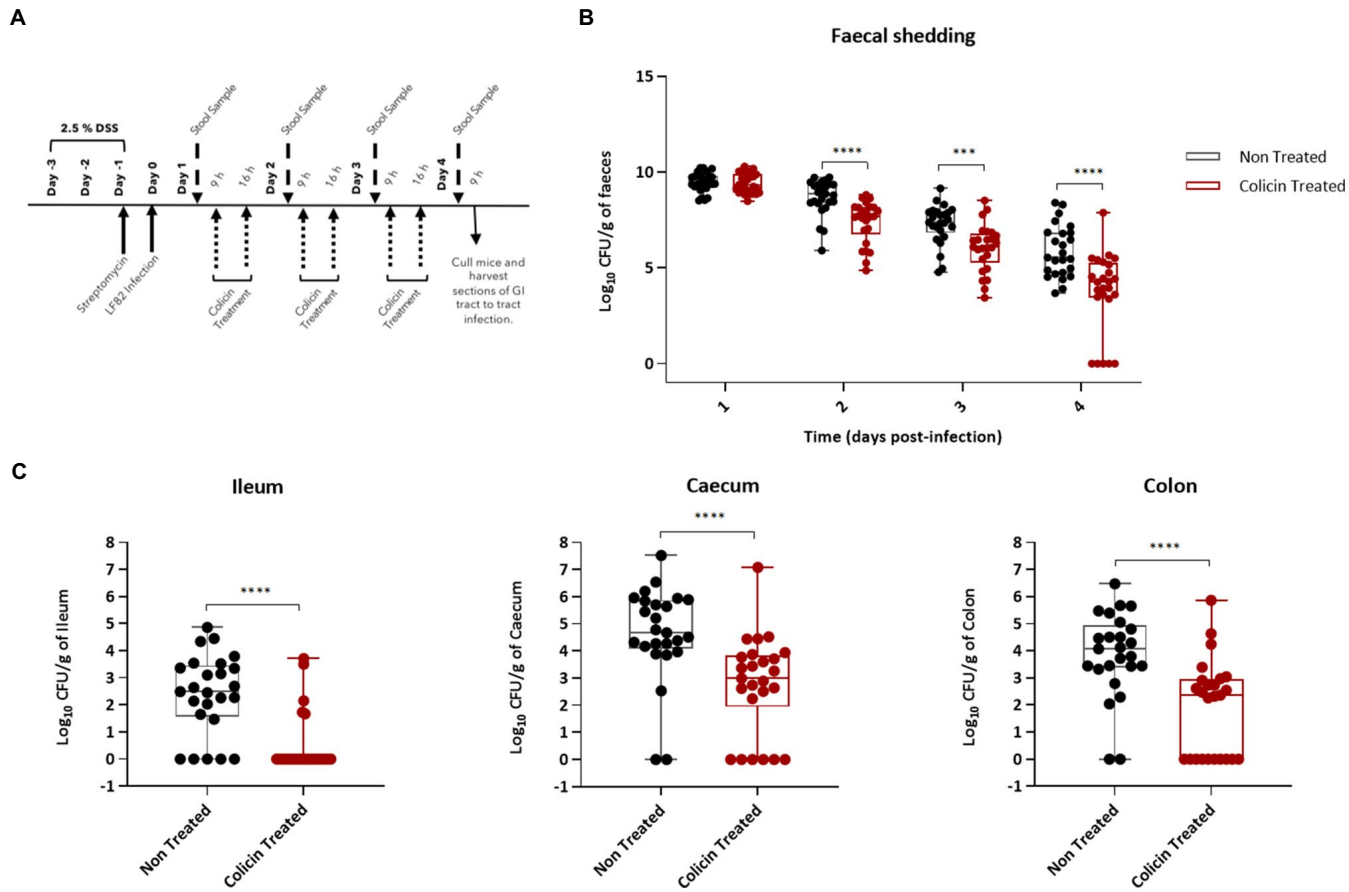
Reduction in LF82 colonization of the lower gastrointestinal (GI) tract in infected mice treated with hydrogel-encapsulated colicins. An *E. coli* LF82 murine infection model was used to assess the efficacy of the hydrogel encapsulated treatment using a combination of E9 and Ia. Day one post-bacterial challenge, mice were treated by oral administration of 200μl of colicin E9 and Ia (dose of 0.5mg each of E9 and Ia) containing hydrogel in a slurry with PBS or 200μl of colicin free hydrogel slurry (control group). **(A)** Experimental scheme. **(B)** Tukey boxplot of fecal shedding of *E. coli* LF82StrpR. **(C)** Levels of LF82StrpR strain in both control (black, *n*=25) and colicin treated (red, *n*=25) groups for the different sections of the GI tract. Statistical analysis was carried out for each subset using a Mann–Whitney test between LF82StrpR infected and colicin-treated groups. ^***^p<0.0004, ^****^p<0.0001.

To determine the ability of colicin microcapsules to reduce *E. coli* levels in the lower GI tract, hydrogel microcapsules were administered twice daily to LF82 colonized mice beginning 1day after LF82 administration (Figure 5A). Mice treated with colicin containing microcapsules shed significantly lower amounts of *E. coli* (median cfu values) in feces compared with the control group, which were administered empty microcapsules, on all days following treatment: day 2 (0.9 log units lower), day 3 (1.2 log units), and day 4 (0.9 log units; Figure 5B). At 4-days post-LF82 administration, mice were killed and LF82 levels in tissue samples from the lower GI tract were determined. Significant decreases in LF82 levels (median cfu values) were found in tissue samples from mice treated with colicin microcapsules in the ileum (2.5 log units), caecum (1.5 log units), and colon (1.7 log units) relative to control mice (Figure 5C). Interestingly, no colonies were isolated in a number of tissue samples and in fecal samples from day 4, indicating that eradication of *E. coli* may be feasible on prolonged colicin treatment.

## Discussion

In this work, we have demonstrated that colicins can be successfully formulated in hydrogel microcapsules in an active form and that formulated colicin is able to reduce *E. coli* levels in a murine colonization model. The membrane emulsification process resulted in controlled fabrication of hydrogel capsules, which were relatively uniform in size and were small enough to be administered *via* oral gavage to mice (Figure 1). The encapsulation process resulted in a high yield of encapsulated colicin and the process of manufacturing the hydrogel capsules did not affect colicin activity upon release. The capsules displayed pH responsive characteristics suitable for targeted delivery of the therapeutic cargo in response to changes in pH (Figure 3). The design of the particles to deliver a slow rate of release allowed delivery of the encapsulated colicin to different parts of the GI tract given the relatively small differences in pH values observed in the mouse lower GI tract (McConnell et al., 2008). For human therapeutic applications, other methacrylate polymers, e.g., L100 (pH 6) or S100 (pH 7) could be used to target delivery in response to more significant differences in pH (Vinner et al., 2019). Polymers which may release encapsulated cargo based on the presence of virulence factors in the environment could potentially be a more sophisticated targeted approach (Bean et al., 2014).

Previous published colicin encapsulation research is limited to encapsulation in pectin hydrogel beads with low reported encapsulation efficiency of ~1%. The encapsulated beads were administered orally to mice, and after 6days of treatment, no significant differences reported in CFU/g fecal matter between treated and non-treated mice (Brown, 2015). This may be attributed to the lack of targeted delivery and limited stability of colicins exposed to the gastric environmental conditions. Membrane emulsification process used in the present study has previously been used for encapsulation of phage biotherapeutics. Encapsulated phages were shown to be released at defined pH values dependent on the type of pH-responsive polymer used in the formulation and phages shown to withstand gastric acid exposure at pH 1.5 for up to 2h (Richards and Malik, 2021).

The polymers used for the microcapsule fabrication are routinely used in food formulations and for enteric delivery applications (Evonik healthcare). The polymers utilized for colicin encapsulation have regulatory approval for healthcare applications and are generally regarded as safe (GRAS) for human consumption by the US FDA. The safety of alginic acid has extensively been researched and recognized as posing no toxicity risks in mammals. JECFA (1993) reviewed alginate toxicology literature and summarized there were no toxic effects when tested in rats at levels up to 13,500mg sodium alginate/kg body weight (bw) per day (JECFA, 1993). No carcinogenic effects were reported at 37,500mg sodium alginate/kg bodyweight per day in mice (Rychen et al., 2017). Furthermore, Eudragit polymers were tested for effects on the nervous system through nanoparticle administration to rats either orally or intraperitoneally. Animals were killed at timepoints up to 3weeks. Histological examination determined a normal histological picture and insignificant changes, thus concluding diminutive toxic effects on the brain (Abdel-Wahhab et al., 2017).

Small rodent animal models are routinely used in preclinical testing of drugs and vaccines due to their size and low cost. pH responsive drug delivery platforms rely on defined changes in physiological pH along the GI tract to trigger release of therapeutic cargo. Encapsulation of therapeutic agents may overcome delivery issues such as degradation of the therapeutic upon exposure to stomach acidity or due to enzymatic activity, and colicins are particularly susceptible to proteolysis-related degradation (Schulz et al., 2015). Polymethacrylate and cellulose-based enteric capsules are routinely used for delivery of drugs or vaccines to the GI tract by dissolving only when the pH of the environment exceeds a threshold level. Knowledge of the gut pH of the mouse is critical in selecting the appropriate polymer for formulation of microcapsules for targeted delivery and controlled release. The residence time of the capsules and the fluid content of the GI tract are other critical factors in targeted delivery of the therapeutic agent at the site of infection. The moderately acidic pH values of the stomach tissue samples measured in the present study were low enough to prevent colicin release from the microcapsules in the stomach. The pH of the small intestine, caecum, and colon tissue samples was found to be higher than pH 5.5 albeit when the diluent used may have influenced the measured pH values. A pH above pH 5 is suitably high for dissolution of the fabricated L100-55/alginate hydrogel microcapsules, resulting in the successful release of the encapsulated colicin cargo in the lower GI tract in the mice. Previous studies in which measurement of mouse GI tract pH was performed indicated variability in measured pH between individual animals with mean values of stomach pH around pH 4, and pH 5 for intestinal tract tissue samples, using undiluted tissue contents (McConnell et al., 2008). Drug formulations are often given to animals by oral gavage with the dose volume determined by the stomach volume of the animal and typically, doses not exceeding 0.4ml are recommended for studies in mice (Wolfensohn and Lloyd, 1994). We used 0.2ml of buffer with suspended hydrogels in this study. Previous studies have reported that very low levels of fluid, less that 1ml, are present within the mouse gastrointestinal tract (McConnell et al., 2008). Noninvasive studies of GI transit times in mice suggest oral administered microcapsules may pass through into the small intestine within 1h of ingestion, with a total transit time of 6h to the colon (Padmanabhan et al., 2013). The *in vitro* data reported here (Figure 3) showed the release kinetics of colicin over a 2h period from the hydrogel microcapsules would be sufficient to allow encapsulated colicin to be released in the lower GI tract. The low water content of the mouse GI tract and the relatively low intestinal pH may further slow the rate of release *in vivo* which may result in a significant dose of the encapsulated colicin cargo being released much lower in the GI tract.

Significant *in vivo* reduction in bacterial counts (cfu/ml) in all GI tract tissue samples suggests that encapsulation of the colicins in the microcapsules could serve as a useful strategy for evaluating the therapeutic potential of protein antibiotics, which may otherwise degrade due to exposure to the harsh environmental conditions in the stomach and along the GI tract. The capacity to administer protein antibiotics to the lower GI tract offers opportunities for the deployment of these narrow-spectrum antibiotics for re-engineering of the gut microbiota through selective targeting of specific bacterial species. This may be particularly useful in decolonization of the gut microbiota of potential pathogens such as drug-resistant resistant Enterobacteriaceae that may be dominant and cause disease.

## Data Availability Statement

The raw data supporting the conclusions of this article will be made available by the authors, without undue reservation.

## Ethics Statement

The animal study was reviewed and approved by University of Glasgow Ethics Committee, License number: Home Office, United Kingdom (PPL60/8797 and P64BCA712).

## Author Contributions

NC, KR, DM, and DW contributed to conception and design of the study and wrote the first draft of the manuscript. NC performed the statistical analysis. All authors contributed to manuscript revision, read, and approved the submitted version.

## Funding

DM acknowledges funding from the UK Engineering and Physical Sciences Research Council (Grant No. EP/M027341/1), “Tackling Antimicrobial Resistance: An Interdisciplinary Approach.” KR acknowledges a Loughborough University PhD studentship. DW acknowledges funding from Scottish Enterprise (Grant No. PS7305CA55), the MRC (Grant No. MC_PC_15039), and the Wellcome Trust (Grant No. 201505/Z/16/Z).

## Conflict of Interest

The authors declare that the research was conducted in the absence of any commercial or financial relationships that could be construed as a potential conflict of interest.

## Publisher’s Note

All claims expressed in this article are solely those of the authors and do not necessarily represent those of their affiliated organizations, or those of the publisher, the editors and the reviewers. Any product that may be evaluated in this article, or claim that may be made by its manufacturer, is not guaranteed or endorsed by the publisher.

## Supplementary Material

Supplementary Figure S1 Particle size distributions of the water-in-oil (W/O) emulsion droplets and the final cross-linked hydrogel microcapsules. The differential volume distributions **(A)** and cumulative volume distributions **(B)** are shown, respectively. Droplets refer to the initial (W/O) emulsion prior to cross-linking (gelation), whereas the particles refer to the final hydrogel microcapsules produced after L100 precipitation due to protonation in TSA and the alginate cross-linking in calcium chloride.

Supplementary Figure S2 Mechanism of encapsulation and pH-triggered release action. The capsules were initially fabricated using membrane emulsification **(A)** the alginate and L100-55 polymer chains form the internal capsule structure. The L100-55 was protonated through incubation in 0.05M TSA+Miglyol +5% (w/v) PGPR. The capsules were then cross-linked using 0.1M CaCl_2_ as the calcium ions cross-link the alginate chains forming an eggbox-like structure. The polymer chains dissociate upon exposure to pH 5.5 and above and the colicin subsequently released. **(B)** L100-55 chemical structure. **(C)** SEM imaging showing Freeze-dried L100-55 microcapsules (left) and Helium Ion Microscopy image (right) of the internal Eudragit-Alginate matrix. A microcapsule having particle size about 10μm was cut in half using a Ne^+^ beam and after 180° rotation imaged with He^+^. A CPD-dried microparticle was milled with the Ne^+^ beam. After 180° rotation, the cut surface was imaged with He^+^. Higher magnification image of the cross section showing internal porous matrix of the polymer. Schematic **(A,B)** created with BioRender.com.

Supplementary Figure S3 Effect of alginate concentration on W/O emulsion droplet characteristics. Emulsions were produced using a membrane with 40μm pores arranged as a ringed array. The dispersed phase was composed of either 0.5, 1, or 2% (w/v) medium viscosity alginate with 10% (w/v) L100-D55 polymer dissolved in dH_2_O. The continuous phase was composed of miglyol +5% (v/v) PGPR. A flow rate of 25ml/h was used along with a stirrer speed of 300 RPM. Emulsions were produced in 55ml batches, 50ml continuous phase +5ml dispersed phase. **(A)** Mean particle diameters are displayed as columns; coefficient of variation values is presented as data points corresponding to the secondary axis. Emulsions were tested on the LS coulter immediately after production. **(B)** Optical images taken immediately after emulsion production using the Nikon Phantom camera and the 10x magnification lens. (i) displays the emulsion produced using 0.5% (w/v) alginate, (ii) 1% (w/v) alginate.

## References

[ref1] Abdel-Wahhab M. A. Joubert O. Khadrawy Y. A. Safar R. El-Nekeety A. A. Ronzani C.. (2017). Preliminary safety assessment of Eudragit® polymers nanoparticles administration in the rat brain. J. Appl. Pharm. Sci. 7, 176–185. doi: 10.7324/JAPS.2017.70726

[ref2] Bean J. E. Alves D. R. Laabei M. Esteban P. P. Thet N. T. Enright M. C.. (2014). Triggered release of bacteriophage K from agarose/hyaluronan hydrogel matrixes by staphylococcus aureus virulence factors. Chem. Mater. 26, 7201–7208. doi: 10.1021/cm503974g

[ref3] Behrens H. M. Lowe E. D. Gault J. Housden N. G. Kaminska R. Moritz Weber T.. (2019). Pyocin S5 import into *Pseudomonas aeruginosa* reveals a generic mode of bacteriocin transport. bioRxiv [Preprint]. doi: 10.1101/856047 PMC7064778 32156826

[ref4] Brown C. L. (2015). The development of colicins as novel antimicrobials against crohn’s disease associated adherent-invasive *Escherichia coli*. PhD thesis. University of Glasgow. Available at: https://eleanor.lib.gla.ac.uk/record=b3153152

[ref5] Brown C. L. Smith K. Wall D. M. Walker D. (2015). Activity of species-specific antibiotics against crohn’s disease-associated adherent-invasive *Escherichia coli*. Inflamm. Bowel Dis. 21, 2372–2382. doi: 10.1097/MIB.0000000000000488, PMID: 26177305

[ref6] Cascales E. Buchanan S. K. Duche D. Kleanthous C. Lloubes R. Postle K.. (2007). Colicin biology. Microbiol. Mol. Biol. Rev. 71, 158–229. doi: 10.1128/MMBR.00036-06, PMID: 17347522 PMC1847374

[ref7] Darfeuille-Michaud A. Boudeau J. Bulois P. Neut C. Glasser A. L. Barnich N.. (2004). High prevalence of adherent-invasive *Escherichia coli* associated with ileal mucosa in crohn’s disease. Gastroenterology 127, 412–421. doi: 10.1053/j.gastro.2004.04.061, PMID: 15300573

[ref8] Holt K. E. Thieu Nga T. V. Thanh D. P. Vinh H. Kim D. W. Vu Tra M. P.. (2013). Tracking the establishment of local endemic populations of an emergent enteric pathogen. Proc. Natl. Acad. Sci. U. S. A. 110, 17522–17527. doi: 10.1073/pnas.1308632110, PMID: 24082120 PMC3808646

[ref9] Jakes K. S. Finkelstein A. (2010). The colicin Ia receptor, Cir, is also the translocator for colicin Ia. Mol. Microbiol. 75, 567–578. doi: 10.1111/j.1365-2958.2009.06966.x, PMID: 19919671 PMC3493618

[ref10] JECFA (1993). Alginic acid and its ammonium; calcium; potassium and sodium salts. (Join FAO/WHO Expert Committee on Food Additives). 755.

[ref11] Kleanthous C. (2010). Swimming against the tide: progress and challenges in our understanding of colicin translocation. Nat. Rev. Microbiol. 8, 843–848. doi: 10.1038/nrmicro2454, PMID: 21060316

[ref12] Lloyd-Price J. Arze C. Ananthakrishnan A. N. Schirmer M. Avila-Pacheco J. Poon T. W.. (2019). Multi-omics of the gut microbial ecosystem in inflammatory bowel diseases. Nature 569, 655–662. doi: 10.1038/s41586-019-1237-9, PMID: 31142855 PMC6650278

[ref13] Logan L. K. Weinstein R. A. (2017). The epidemiology of carbapenem-resistant enterobacteriaceae: the impact and evolution of a global menace. J. Infect. Dis. 215, S28–S36. doi: 10.1093/infdis/jiw282, PMID: 28375512 PMC5853342

[ref14] McCaughey L. C. Ritchie N. D. Douce G. R. Evans T. J. Walker D. (2016). Efficacy of species-specific protein antibiotics in a murine model of acute *Pseudomonas aeruginosa* lung infection. Sci. Rep. 6:30201. doi: 10.1038/srep30201, PMID: 27444885 PMC4957109

[ref15] McConnell E. L. Basit A. W. Murdan S. (2008). Measurements of rat and mouse gastrointestinal pH, fluid and lymphoid tissue, and implications for in-vivo experiments. J. Pharm. Pharmacol. 60, 63–70. doi: 10.1211/jpp.60.1.0008, PMID: 18088506

[ref16] Nedialkova L. P. Denzler R. Koeppel M. B. Diehl M. Ring D. Wille T.. (2014). Inflammation fuels colicin Ib-dependent competition of Salmonella serovar Typhimurium and *E. coli* in enterobacterial blooms. PLoS Pathog. 10:e1003844. doi: 10.1371/journal.ppat.1003844, PMID: 24391500 PMC3879352

[ref17] Padmanabhan P. Grosse J. Asad A. B. M. A. Radda G. K. Golay X. (2013). Gastrointestinal transit measurements in mice with 99mTc-DTPA-labeled activated charcoal using NanoSPECT-CT. EJNMMI Res. 3:60. doi: 10.1186/2191-219X-3-60, PMID: 23915679 PMC3737085

[ref18] Pommer A. J. Cal S. Keeble A. H. Walker D. Evans S. J. Kühlmann U. C.. (2001). Mechanism and cleavage specificity of the H-N-H endonuclease colicin E9. J. Mol. Biol. 314, 735–749. doi: 10.1006/jmbi.2001.5189, PMID: 11733993

[ref19] Ravi A. Halstead F. D. Bamford A. Casey A. Thomson N. M. Van Schaik W.. (2019). Loss of microbial diversity and pathogen domination of the gut microbiota in critically ill patients. Microb. Genomics 5:e000293. doi: 10.1099/mgen.0.000293, PMID: 31526447 PMC6807385

[ref20] Richards K. Malik D. J. (2021). Microencapsulation of bacteriophages using membrane emulsification in different pH-triggered controlled release formulations for oral administration. Pharmaceuticals 14:424. doi: 10.3390/ph14050424, PMID: 34063218 PMC8147480

[ref21] Rychen G. Aquilina G. Azimonti G. Bampidis V. Bastos M. L. Bories G.. (2017). Safety and efficacy of sodium and potassium alginate for pets, other non food-producing animals and fish. EFSA J. 15:e04945. doi: 10.2903/j.efsa.2017.4945, PMID: 32625597 PMC7009951

[ref22] Schulz S. Stephan A. Hahn S. Bortesi L. Jarczowski F. Bettmann U.. (2015). Broad and efficient control of major foodborne pathogenic strains of *Escherichia coli* by mixtures of plant-produced colicins. Proc. Natl. Acad. Sci. U. S. A. 112, E5454–E5460. doi: 10.1073/pnas.1513311112, PMID: 26351689 PMC4603501

[ref23] Six A. Mosbahi K. Barge M. Kleanthous C. Evans T. Walker D. (2020). Pyocin efficacy in a murine model of *Pseudomonas aeruginosa* sepsis. bioRxiv [Preprint]. doi: 10.1101/2020.03.27.011908 PMC8361349 34142136

[ref24] Vinner G. K. Richards K. Leppanen M. Sagona A. P. Malik D. J. (2019). Microencapsulation of enteric bacteriophages in a pH-responsive solid oral dosage formulation using a scalable membrane emulsification process. Pharmaceutics 11:475. doi: 10.3390/pharmaceutics11090475, PMID: 31540028 PMC6781335

[ref25] Wadolkowski E. A. Laux D. C. Cohen P. S. (1988). Colonization of the streptomycin-treated mouse large intestine by a human fecal *Escherichia coli* strain: role of adhesion to mucosal receptors. Infect. Immun. 56, 1036–1043. doi: 10.1128/iai.56.5.1036-1043.1988, PMID: 2833441 PMC259758

[ref26] Wolfensohn S. Lloyd M. (1994). Handbook of Laboratory Animal Management. Oxford: Oxford University Press.

