# Supplementary figures and images for: Targeted Delivery of Narrow-Spectrum Protein Antibiotics to the Lower Gastrointestinal Tract in a Murine Model of Escherichia coli Colonization
Source: Front Microbiol. 2021 Oct 14;12:670535. doi: 10.3389/fmicb.2021.670535 (PMC8551963; doi:10.3389/fmicb.2021.670535)

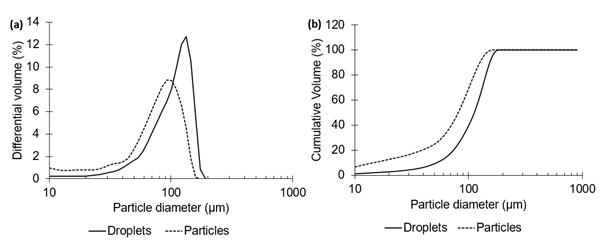

Supplement: Supplementary Figure S1 — Particle size distributions of the water-in-oil (W/O) emulsion droplets and the final cross-linked hydrogel microcapsules. The differential volume distributions (A) and cumulative volume distributions (B) are shown, respectively. Droplets refer to the initial (W/O) emulsion prior to cross-linking (gelation), whereas the particles refer to the final hydrogel microcapsules produced after L100 precipitation due to protonation in TSA and the alginate cross-linking in calcium chloride. [file Image_1.TIF]

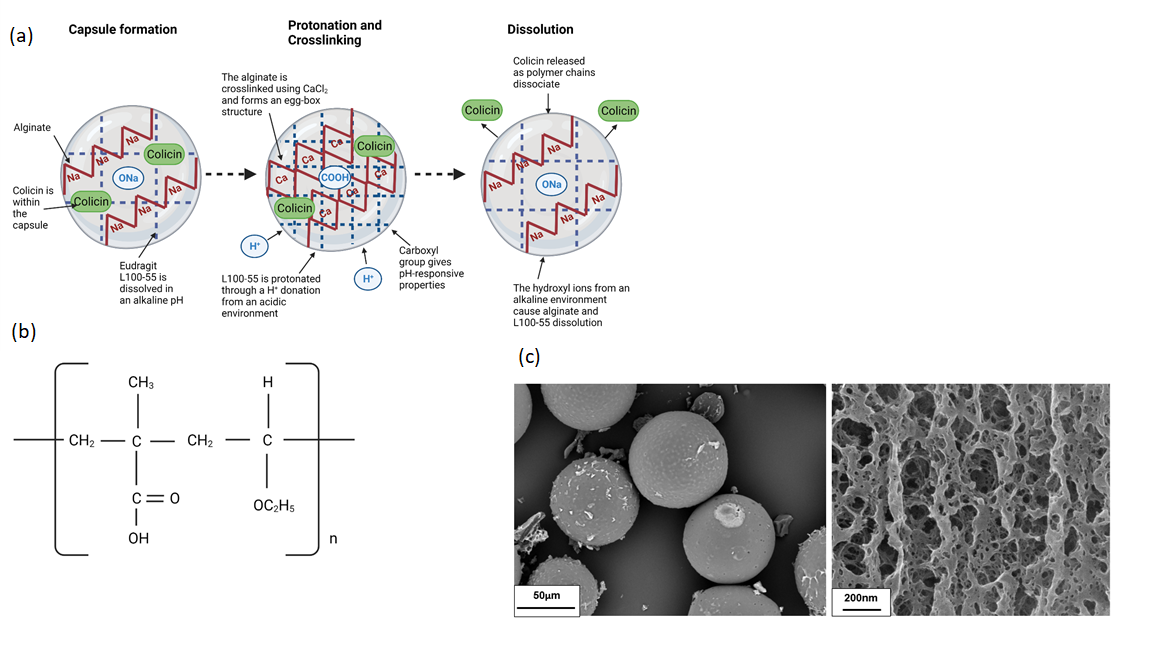

Supplement: Supplementary Figure S2 — Mechanism of encapsulation and pH-triggered release action. The capsules were initially fabricated using membrane emulsification (A) the alginate and L100-55 polymer chains form the internal capsule structure. The L100-55 was protonated through incubation in 0.05M TSA+Miglyol +5% (w/v) PGPR. The capsules were then cross-linked using 0.1M CaCl2 as the calcium ions cross-link the alginate chains forming an eggbox-like structure. The polymer chains dissociate upon exposure to pH 5.5 and above and the colicin subsequently released. (B) L100-55 chemical structure. (C) SEM imaging showing Freeze-dried L100-55 microcapsules (left) and Helium Ion Microscopy image (right) of the internal Eudragit-Alginate matrix. A microcapsule having particle size about 10μm was cut in half using a Ne+ beam and after 180° rotation imaged with He+. A CPD-dried microparticle was milled with the Ne+ beam. After 180° rotation, the cut surface was imaged with He+. Higher magnification image of the cross section showing internal porous matrix of the polymer. Schematic (A,B) created with BioRender.com. [file Image_2.TIF]

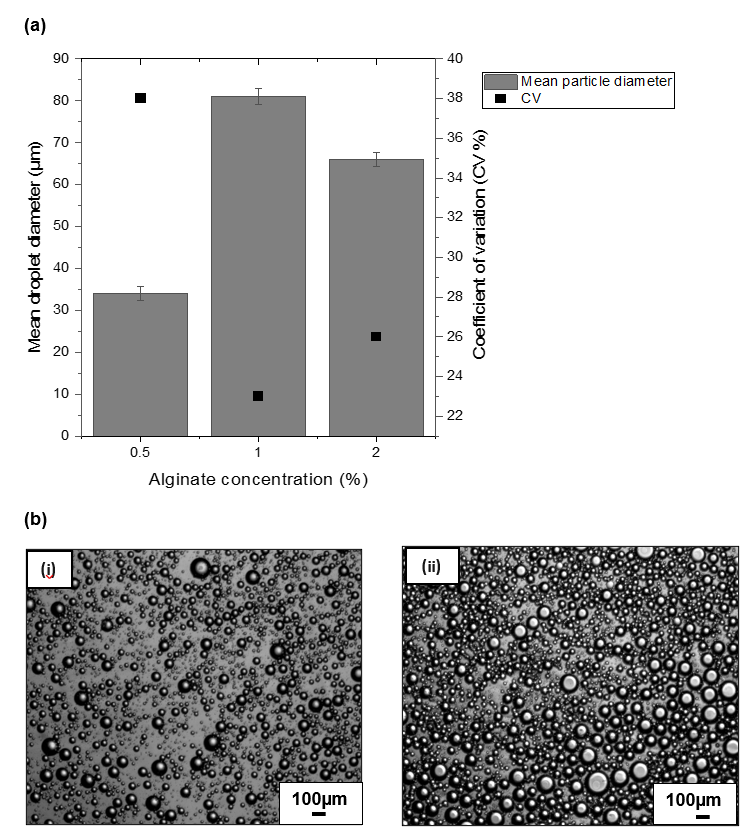

Supplement: Supplementary Figure S3 — Effect of alginate concentration on W/O emulsion droplet characteristics. Emulsions were produced using a membrane with 40μm pores arranged as a ringed array. The dispersed phase was composed of either 0.5, 1, or 2% (w/v) medium viscosity alginate with 10% (w/v) L100-D55 polymer dissolved in dH2O. The continuous phase was composed of miglyol +5% (v/v) PGPR. A flow rate of 25ml/h was used along with a stirrer speed of 300 RPM. Emulsions were produced in 55ml batches, 50ml continuous phase +5ml dispersed phase. (A) Mean particle diameters are displayed as columns; coefficient of variation values is presented as data points corresponding to the secondary axis. Emulsions were tested on the LS coulter immediately after production. (B) Optical images taken immediately after emulsion production using the Nikon Phantom camera and the 10x magnification lens. (i) displays the emulsion produced using 0.5% (w/v) alginate, (ii) 1% (w/v) alginate. [file Image_3.TIF]
